# Cardiopulmonary Exercise Testing to Troubleshoot Rate-Responsive Pacing in an Athlete With Acquired Sinus Node Dysfunction

**DOI:** 10.1016/j.jaccas.2025.106502

**Published:** 2025-12-17

**Authors:** Shreyas Venkataraman, Krasimira Mikhova, Douglas Hall, Nathan Bekele, Daniel H. Cooper, Mustafa Husaini

**Affiliations:** Washington University in Saint Louis, St Louis, Missouri, USA

**Keywords:** cardiac pacemaker, electrophysiology, exercise, treatment

## Abstract

**Background:**

Athletes with sinus node isolation can become fully dependent on sensor-driven pacing. Default accelerometer settings may underperform during exertion.

**Case Summary:**

A 73-year-old former collegiate athlete developed exertional dyspnea after Cox Maze IV procedure; postoperative testing showed sinus node isolation, and he received a dual-chamber pacemaker. A cardiopulmonary exercise test (CPET) revealed a blunted chronotropic response, with his heart rate plateauing at 105 beats/min (device activity setpoint). A repeat CPET with real-time programming (lower activity threshold, higher activities of daily living rate, rate-adaptive atrioventricular delay/limits) restored rate escalation within weeks, with symptom resolution and peak oxygen consumption volume improving from 87% to 102% predicted.

**Discussion:**

CPET can both diagnose device-mediated chronotropic limitation and provide a physiologic target for on-table pacemaker programming in highly active patients, where activity sensors often miss intermittent workloads.

**Take-Home Message:**

This case underscores the limitations of activity-based sensors in athletes and highlights the utility of CPET in diagnosing and personalizing pacemaker programming to restore functional capacity.

## Visual Summary

**Figure:**
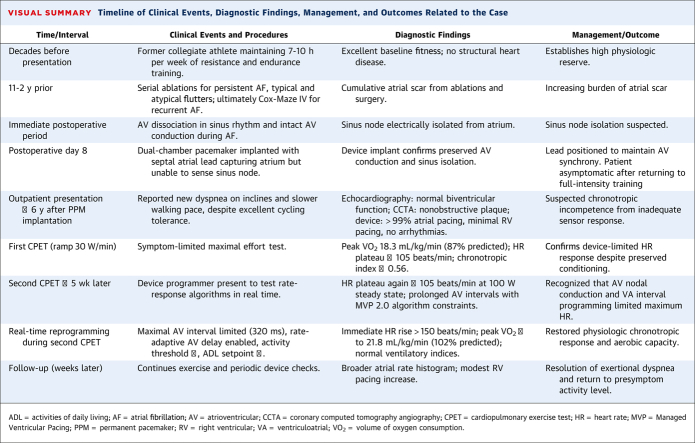

## History of Present Illness

An active 73-year-old former collegiate football player with a history of atrial arrhythmias and pacemaker dependence presented for follow-up reporting shortness of breath with specific types of exertion. Although he was comfortable with hour-long cycling, he felt disproportionately dyspneic on inclines and unable to keep pace walking with his sedentary friends.

**Figure 1:**
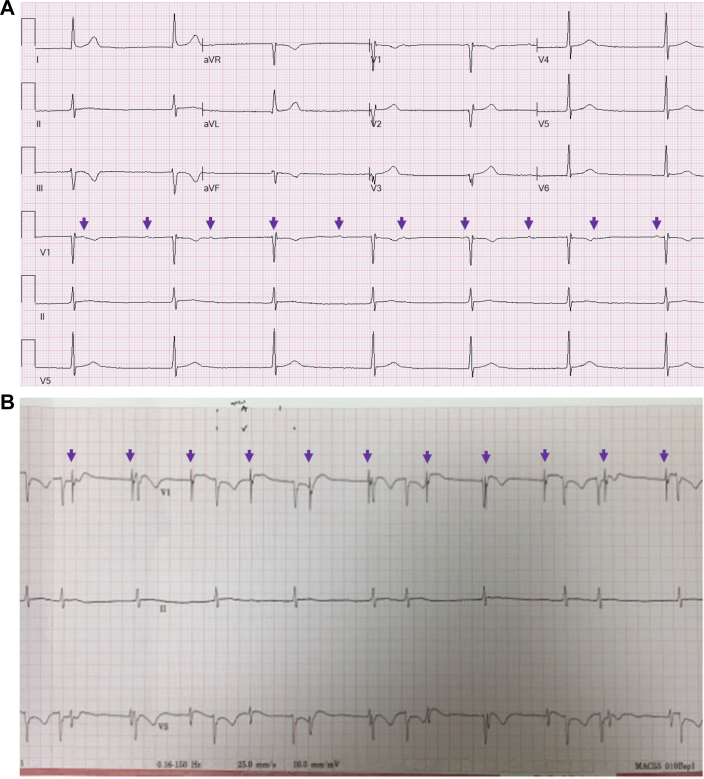
Electrocardiograms After Cox Maze IV Procedure Postoperative ECG (A) reveals low-amplitude P waves with AV dissociation and a junctional escape. (B) Atrial ECG, with leads V_1_ and V_5_ connected to the epicardial atrial pacing lead and lead II as conventional surface tracing, show AV dissociation with PVCs. Purple arrows denote atrial activity. AV = atrioventricular; ECG = electrocardiogram; PVC = premature ventricular contraction.

**Figure 2:**
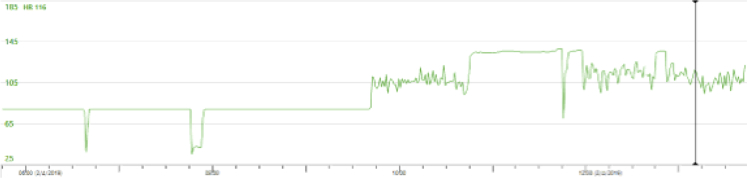
Telemetry Trend Telemetry showing periods of tachycardia (when atrial rhythm is atrial fibrillation) and bradycardia (when atrial rhythm is sinus rhythm not conducting to ventricles).

He previously underwent typical atrial flutter ablation (11 years prior), persistent atrial fibrillation (AF) ablation (7 years prior), and atypical left atrial flutter ablation (6 years prior), followed by Cox-Maze IV procedure^1^ in 2019 for recurrent symptomatic persistent AF. Postoperatively, he demonstrated atrioventricular (AV) dissociation in sinus rhythm (Figure 1). However, he continued to have paroxysmal AF, while demonstrating intact AV conduction with a rapid ventricular response (Figure 2) consistent with sinus node isolation as a result of his underlying atriopathy and multiple extensive ablations. This was confirmed during dual-chamber pacemaker placement on postoperative day 8; a septal atrial lead captured in the atrium and conducted to the ventricle with a narrow QRS but the lead could not sense sinus activity at this location. Given intact AV conduction, his atrial lead was secured to this position to provide mechanical atrial systole and minimize ventricular pacing.

The patient maintained a decades-long, high-volume exercise regimen and continued to train effectively. Vital signs in clinic were notable for a heart rate of 50 beats/min. Examination revealed a physically fit man with a normal cardiopulmonary examination.

## Past Medical History

The patient's medical history included atrial arrhythmias and sinus node isolation requiring dual-chamber pacemaker, along with hypertension and asthma.

## Differential Diagnosis

Considerations included recurrent atrial arrhythmia, coronary artery disease, and heart failure, and—given sinus node isolation with pacemaker dependence to drive his heart rate response to exercise—the differential diagnosis in this case also included inadequate rate response to physical exertion.

## Investigations

Transthoracic echocardiography demonstrated normal biventricular size and function and moderate right atrial dilation. Coronary computed tomography angiography indicated nonobstructive plaque. Pacemaker interrogation of the Medtronic dual-chamber device programmed in MVP (Managed Ventricular Pacing) mode (AAIR⇔DDDR) indicated a lower rate limit of 50 beats/min, upper rate limit of 160 beats/min, and no atrial arrhythmia events. He was 99.9% paced in the atrium and 2.7% paced in the right ventricle.

**Figure 3:**
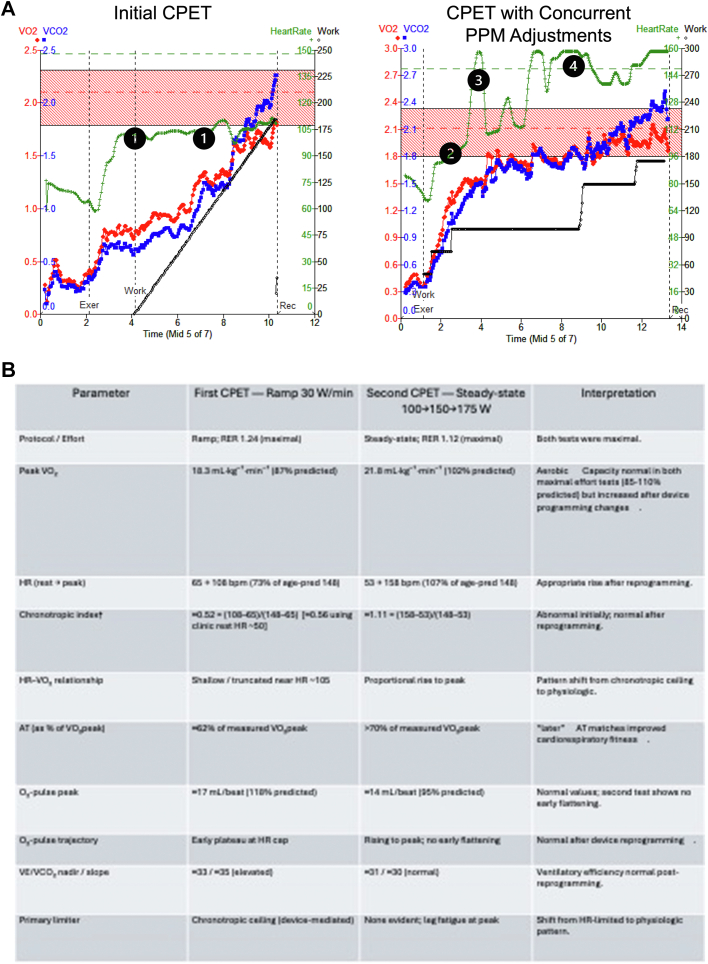
Cardiopulmonary Exercise Stress Test to Guide PPM Adjustments to Improve Exercise Intolerance (A) Initial CPET and (B) subsequent CPET 5 weeks later with concurrent device programming. The dashed green line indicates the age-predicted maximal heart rate; the dashed red line represents the predicted peak VO_2_, with the shaded red zone indicating 85% to 110% of predicted. The initial CPET demonstrated a peak VO_2_ of 18.3 mL/kg/min (87% predicted) and anaerobic threshold occurring at 62% of peak VO_2_, which was lower than expected for the patient's training history. Given his HR plateaued around 105 beats/min at a workload of ∼100 W (1), the plan was made to repeat CPET a few weeks later with a device programmer present to make real-time adjustments to his device. After a short warm-up period, the patient reached 100 W and entered steady-state exercise with HR ∼105 beats/min (2). Enabling a maximum AV interval limit and rate-adaptive AV delays, as well as adjustments to activity thresholds, allowed for a more appropriate increase in heart rate (3). The ability to more readily switch modes to DDDR allowed the patient to maintain his heart rate with exertion (4). This CPET demonstrated a peak VO_2_ of 21.8 mL/kg/min (102% predicted) and RER of 1.12, with anaerobic threshold >70% of peak VO_2_, correlating with immediate resolution of exertional symptoms. (Bottom) Table comparing key indices from the 2 CPETs. AV = atrioventricular; CPET = cardiopulmonary exercise test; HR = heart rate; PPM = permanent pacemaker; RER = respiratory exchange ratio; VO_2_ = volume of oxygen consumption.

The patient underwent a cardiopulmonary exercise test (CPET) with a 30 W/min ramp protocol and achieved a maximal test (respiratory exchange ratio: 1.24). Peak volume oxygen consumption (VO_2_) was 18.3 mL/kg/min (87% predicted), with anerobic threshold at 62% of the measured VO_2_, indicating a reasonable level of aerobic conditioning (Figure 3A). Of note, the patient's heart rate plateaued at 105 beats/min despite increasing workload, yielding a chronotropic index of ∼0.56 (normal, often 0.8-1.3).

## Management

With no traditional etiology for the patient's exertional symptoms, attention was focused on his pacemaker's rate-adaptive response. The hypothesis was that his device was not responding quickly enough to meet the metabolic demands of his body during strenuous exercise such as climbing hills, and thus changes were made to augment its response. Given discordance between fitness history and VO_2_ performance, the plan was made to perform repeat CPET with a steady-state protocol of 100 W with concurrent pacemaker adjustments. Two additional CPETs were performed approximately 5 weeks apart, minimizing the likelihood of a meaningful training effect because the patient's exercise regimen remained stable. He exercises 7 to 10 hours per week—typically 35 to 50 minutes of resistance training 5 to 6 times weekly and 30 to 45 minutes of interval aerobic training 3 to 4 times weekly—and has maintained this routine for decades since college. He did not initiate any new training program or increase workload during the interval.

During his steady-state CPET, the patient's heart rate again stabilized around 105 beats/min, which was determined to be a result of decremental AV nodal conduction and programming details of the Medtronic MVP algorithm. The MVP algorithm, designed to minimize ventricular pacing, switches from AAIR to DDDR only in the event of loss of conduction, and the MVP 2.0 algorithm has no default maximum AV interval. Furthermore, the minimum ventriculoatrial intervals are limited to 40% of the sensor-indicated rate. For instance, if the device's rate response indicated a desired rate of 120 beats/min (corresponding to a cycle length of 500 ms), the time between a V-sensed event and subsequent atrial pacing must be at least 200 ms. This restriction, in combination with significant AV interval prolongation and decremental AV conduction with exercise, was limiting the atrial pacing rate.

With the assistance of a device representative, the patient's pacemaker was adjusted while monitoring his heart rate and symptoms (Table 4). A maximum AV interval limit was set at 320 ms, and the rate-adaptive AV feature was also enabled, limiting the paced AV interval to 140 ms with elevated heart rates. These changes increased the right ventricular pacing burden but allowed the patient to achieve higher heart rates. The amount of activity sensed by the device needed to elicit rate response was also tuned by changing the activity threshold from medium-low to low and the activities of daily living setpoint from 7 to 15.

## Follow-Up

After these programming adjustments, a repeat CPET employing a steady-state protocol demonstrated an improvement in exercise capacity. The peak VO_2_ was at 102% of predicted (21.8 mL/kg/min), and anaerobic threshold was at >70% of measured VO_2_ (Figure 3B). The patient's heart rate rose from 53 to 158 beats/min (107% of age-predicted maximum), resulting in a chronotropic index of ∼1.10 (normal: 0.8-1.3).

**Figure 4:**
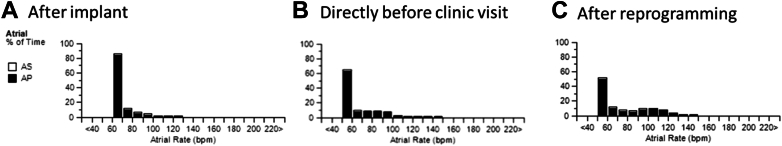
Rate Histograms on Device Interrogation The atrial rate was less skewed with more aggressive rate-responsive programming, showing less time spent around the lower rate limit.

Repeat device interrogation showed an improvement in rate responsiveness with broader atrial and ventricular rate histograms (Figure 4). The patient's right ventricular pacing burden increased from 2.7% to 27.3% as a result of decreasing his AV intervals. This does increase his risk for developing adverse effects from pacing, including pacing-induced cardiomyopathy; reassuringly, he has not manifested any symptoms or preclinical evidence of this. At the time of device implantation, his anticipated ventricular pacing burden was low, and left bundle branch area pacing was not regularly performed at our institution. Longitudinal follow-up will be important in order to monitor for deleterious effects of chronic right ventricular pacing, at which point further programming changes or a device upgrade could be considered.

The patient reported near-complete resolution of his symptoms and could climb multiple flights of stairs without dyspnea or fatigue—a level of exertion that had previously been limiting. This improvement was attributed to enhanced chronotropic response. These findings reinforce the effectiveness of CPET-guided device optimization in restoring functional performance in highly active patients reliant on rate-responsive pacing rather than ventilatory or peripheral factors.

## Discussion

The sinus node lies at the junction of the anterolateral superior vena cava and the right atrium, lacks discrete borders, and extends from the subendocardium to the subepicardium, making it somewhat resistant to injury during endocardial ablation. While superior vena cava–based triggers are occasionally targeted during AF ablation, the right-sided endocardial ablation lesions in our patient were at distant sites in the cavotricuspid isthmus and coronary sinus. Nevertheless, the lines of conduction block created by endocardial radiofrequency ablation, underlying atrial fibrosis, and the Cox Maze surgical ablation lesion set resulted in sinus node isolation. As a result, a pacemaker lead in the sinus node region would be able to sense his native sinus node activity, but pacing at this location would not result in mechanical atrial systole or conduct to the ventricles. Thus, the atrial lead was placed in a septal location, to preserve mechanical AV synchrony and native conduction, at the expense of being blinded to his sinus rate.

During exercise, cardiac output increases via stroke volume and heart rate in response to increased sympathetic tone and circulating catecholamines. These CPET findings justified device reprogramming—lowering the activity threshold and tuning rate-adaptive pacing and MVP—to hasten the heart rate response and restore exercise tolerance. In rate-adaptive pacing, a sensor is needed to prompt this heart rate response.^2^^,^^3^ Two commercially available sensors include activity accelerometers and impedance sensors that estimate minute ventilation or ventricular contractility (Table 1). Each can misclassify effort and produce nonphysiologic rates; typical scenarios are listed in Table 2. Blended sensors combine a physiologic parameter (minute ventilation) with an activity sensor to overcome single-sensor limitations. Prospective trials have not shown a benefit to this strategy; one study showed improved chronotropic response with a blended sensor compared with activity sensor alone, without quality of life improvements.^4^ Lack of individualized programming was cited as a limitation.

**Table 1:** Programming Changes Made During CPET

	Maximum AV Interval	Rate-Adaptive AV Interval	Activity Threshold	ADL Setpoint
Before CPET	None	Disabled	Medium-low	7
After CPET	320 ms	Enabled	Low	15
Effect	Limited the maximum AV interval so the device will pace in the event of significant AV delay	Further shortens the AV interval (as short as 140 ms) at faster heart rates	Decreases the amount of activity needed to trigger the rate-responsiveness sensor	Increased the amount of activity needed to trigger ADL rate

ADL = activities of daily living; AV = atrioventricular; CPET = cardiopulmonary exercise test.

**Table 2:** Clinically Available Strategies for Rate-Adaptive Pacing

Physiologic Parameter	Sensor	Physiologic Basis	Manufacturer
Activity	Vibration/acceleration	Exercise results in change in velocity and direction of acceleration, sensed by accelerometer within the device.	All (Abbott, Biotronik, Boston Scientific, Medtronic)
Minute ventilation	Transthoracic impedance	Exercise results in increased minute ventilation, which is proportional to heart rate until the anaerobic threshold is reached. Minute ventilation is approximated by frequently sampling of transthoracic impedance (which increases with inspiration and decreases with expiration).	Boston Scientific
Ventricular contractility	Intracardiac impedance	Exercise results in increased catecholamine stimulation, resulting in increased ventricular stroke volume and contractility. Right ventricular impedance is lower in diastole than in systole, and the slope of impedance change correlates with contractility. The pacing rate is adapted to the contractility in a closed-loop system.	Biotronik

**Figure 5:**
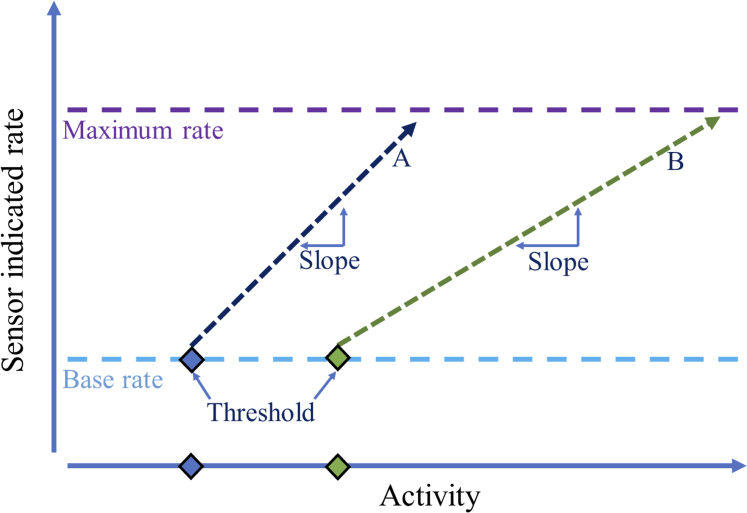
Framework for Understanding Basic Programmable Parameters in Activity Sensor Response The velocity with which the pacing rate increases and decreases to reach the sensor-indicated rate is also programmable. Depending on the manufacturer, additional parameters and activity zones exist. In an athlete, a higher threshold and a lower slope (ie, moving the line from A and B) may be favorable.^2^

Our patient had a device with an activity sensor to prompt rate response. Reprogramming targeted shorter AV intervals and lower thresholds for right ventricular pacing during exercise. This permitted a more physiologic increase in heart rate during exercise, allowing for improved exercise tolerance. Although every manufacturer has different names for programmable features for rate-responsive pacing, commonalities exist (Table 3). In athletes, a higher sensor trigger and a more gradual slope of response (Figure 5) may be preferred to minimize reacting to nonsustained activity and to prevent reaching the maximum rate too quickly.^2^ Successful optimization in our case was achieved with iterative titration of parameters during subsequent clinic visits, where a CPET allowed for immediate and targeted adjustments based on the real-time physiological responses to exercise, a strategy that proved crucial in achieving the desired outcome (Figure 3).

**Table 3:** Clinical Scenarios That Can Result in Inappropriately High or Low Pacemaker Rate Response

Sensed Parameter	Inappropriately High Rates	Inadequate Rate Response
Activity	Environmental vibration (driving over a rough road, use of vibrating machinery)	Stationary exercise, upper limb exertion. Activity sensing is generally more responsive to speed of exercise rather than incline.
Minute ventilation	Upper limb exertion, hyperventilation, positive pressure ventilation, electromagnetic interference (including some ECGs, electrocautery, MRI)	Slower speed of rate response than activity sensors
Ventricular contractility	Postural changes can result in inappropriate tachycardia in some patients	Decreased right ventricular contractility (right ventricular dysfunction, drugs that inhibit inotropy such as beta-blockers, myocardial ischemia)

ECG = electrocardiogram; MRI = magnetic resonance imaging.

**Table 4:** Common Programmable Features to Guide Activity Sensor Response

Programmable Parameter	Name Depending on Manufacturer
How much activity is necessary to trigger the sensor	Threshold, sensor threshold, activity threshold
The relationship between level of activity and sensor-indicated pacing rate	Slope, sensor gain, response factor, exertion response
How quickly the sensor-indicated rate occurs	Reaction time, rate increase, activity acceleration
How quickly during recovery the pacing rate returns to baseline	Recovery time, rate decrease, activity deceleration
Maximum rate allowed by pacing.	Upper sensor rate, maximum sensor rate, maximum activity rate

Given maximal effort on both CPETs as quantified by respiratory exchange ratios >1.1, an unchanged training regimen, a 5-week interval between tests, and the procedural need for a steady-state protocol to enable real-time device changes, the increase in peak VO_2_ from 18.3 to 21.8 mL/kg/min (from 87% to 102% predicted) is very likely most consistent with the effects of device reprogramming.

## Conclusions

We report a case of an athlete with acquired sinus node dysfunction who developed significant exertional intolerance after extensive atrial ablations and Cox Maze IV surgery. With the sinus node electrically isolated, his physiologic chronotropic response became entirely dependent on his pacemaker. Despite appropriate device function, his accelerometer-based rate-responsive pacing failed to adequately match his metabolic demands during exertion, particularly during activities involving incline or intermittent effort.

CPET served as both a diagnostic and interventional tool, revealing suboptimal heart rate response and guiding real-time programming adjustments. Iterative tuning of rate-responsive parameters resulted in immediate and sustained improvements in functional capacity. This case illustrates the limitations of default device programming in athletic patients and highlights the utility of CPET as a powerful, personalized strategy for restoring physiologic pacing.

**Visual Summary:** Timeline of Clinical Events, Diagnostic Findings, Management, and Outcomes Related to the Case

Time/Interval	Clinical Events and Procedures	Diagnostic Findings	Management/Outcome
Decades before presentation	Former collegiate athlete maintaining 7-10 h per week of resistance and endurance training.	Excellent baseline fitness; no structural heart disease.	Establishes high physiologic reserve.
11-2 y prior	Serial ablations for persistent AF, typical and atypical flutters; ultimately Cox-Maze IV for recurrent AF.	Cumulative atrial scar from ablations and surgery.	Increasing burden of atrial scar
Immediate postoperative period	AV dissociation in sinus rhythm and intact AV conduction during AF.	Sinus node electrically isolated from atrium.	Sinus node isolation suspected.
Postoperative day 8	Dual-chamber pacemaker implanted with septal atrial lead capturing atrium but unable to sense sinus node.	Device implant confirms preserved AV conduction and sinus isolation.	Lead positioned to maintain AV synchrony. Patient asymptomatic after returning to full-intensity training
Outpatient presentation ∼6 y after PPM implantation	Reported new dyspnea on inclines and slower walking pace, despite excellent cycling tolerance.	Echocardiography: normal biventricular function; CCTA: nonobstructive plaque; device: >99% atrial pacing, minimal RV pacing, no arrhythmias.	Suspected chronotropic incompetence from inadequate sensor response.
First CPET (ramp 30 W/min)	Symptom-limited maximal effort test.	Peak VO_2_ 18.3 mL/kg/min (87% predicted); HR plateau ∼105 beats/min; chronotropic index ∼0.56.	Confirms device-limited HR response despite preserved conditioning.
Second CPET ∼5 wk later	Device programmer present to test rate-response algorithms in real time.	HR plateau again ∼105 beats/min at 100 W steady state; prolonged AV intervals with MVP 2.0 algorithm constraints.	Recognized that AV nodal conduction and VA interval programming limited maximum HR.
Real-time reprogramming during second CPET	Maximal AV interval limited (320 ms), rate-adaptive AV delay enabled, activity threshold ↓, ADL setpoint ↑.	Immediate HR rise >150 beats/min; peak VO_2_ ↑ to 21.8 mL/kg/min (102% predicted); normal ventilatory indices.	Restored physiologic chronotropic response and aerobic capacity.
Follow-up (weeks later)	Continues exercise and periodic device checks.	Broader atrial rate histogram; modest RV pacing increase.	Resolution of exertional dyspnea and return to presymptom activity level.

ADL = activities of daily living; AF = atrial fibrillation; AV = atrioventricular; CCTA = coronary computed tomography angiography; CPET = cardiopulmonary exercise test; HR = heart rate; MVP = Managed Ventricular Pacing; PPM = permanent pacemaker; RV = right ventricular; VA = ventriculoatrial; VO_2_ = volume of oxygen consumption.

## Funding Support and Author Disclosures

Dr Cooper reports advisory board/consulting with Medtronic and Boston Scientific and fellow teaching conferences with Abbott. All other authors have reported that they have no relationships relevant to the contents of this paper to disclose.

**Take-Home Messages**

• CPET can serve a role in the diagnosis and real-time intervention of chronotropic incompetence in patients with pacemaker-dependent sinus node dysfunction to fine tune parameters.
• In athletes with pacemaker-dependent sinus node isolation, it remains important to identify the limitations of activity-based rate-responsive sensors.
